# Are women with history of pre-eclampsia starting a new pregnancy in good nutritional status in South Africa and Zimbabwe?

**DOI:** 10.1186/s12884-018-1885-z

**Published:** 2018-06-15

**Authors:** Gabriela Cormick, Ana Pilar Betrán, Janetta Harbron, Tina Dannemann Purnat, Catherine Parker, David Hall, Armando H. Seuc, James M. Roberts, José M. Belizán, G. Justus Hofmeyr, on behalf of the Calcium and Pre-eclampsia Study Group, Emilia Makaza, Eunice Tahuringana, Bothwell Guzha, Catherine Parker, Gift Phoramphai, Patience Moloi, Annemarie Greef, Saadiqa Alie, Xoliswa Williams, Pamela Njikelana, Erika von Papendorp, Nicole Minckas, Ricardo López, Paula Rubinstein

**Affiliations:** 1 Instituto de Efectividad Clínica y Sanitaria (IECS-CONICET), Emilio Ravignani, 2024 Buenos Aires, Argentina; 2 Division of Human Nutrition, Department of Human Biology, Faculty of Health Sciences, University of Cape Town, Cape Town, South Africa; 3 HRP – UNDP/UNFPA/UNICEF/WHO/World Bank Special Programme of Research, Development and Research Training in Human Reproduction, Department of Reproductive Health and Research, World Health Organization, Geneva, Switzerland; 4 WHO Regional Office for Europe, World Health Organization, Copenhagen, Denmark; 5 Effective Care Research Unit, University of the Witwatersrand, Johannesburg, South Africa; 6 Effective Care Research Unit, Eastern Cape Department of Health Walter Sisulu University, Mthatha, South Africa; 7 Effective Care Research Unit, University of Fort Hare, East London, South Africa; 8 Department of Obstetrics and Gynaecology, Stellenbosch University and Tygerberg Hospital, Cape Town, South Africa; 9 Epidemiología y Microbiología La Habana, Instituto Nacional de Higiene, Havana, Cuba; 10 Magee-Womens Research Institute, Department of Obstetrics and Gynecology, University of Pittsburgh, Pittsburgh, USA

**Keywords:** Nutrient intake, Weight, Pregnancy, Supplement, Obesity, BMI

## Abstract

**Background:**

Maternal nutritional status before and during pregnancy is an important contributor to pregnancy outcomes and early child health. The aim of this study was to describe the preconceptional nutritional status and dietary intake during pregnancy in high-risk women from South Africa and Zimbabwe.

**Methods:**

This is a prospective observational study, nested to the CAP trial. Anthropometric measurements before and during pregnancy and dietary intake using 24-h recall during pregnancy were assessed. The Intake Distribution Estimation software (PC-SIDE) was used to evaluate nutrient intake adequacy taking the Estimated Average Requirement (EAR) as a cut-off point.

**Results:**

Three hundred twelve women who had pre-eclampsia in their last pregnancy and delivered in hospitals from South Africa and Zimbabwe were assessed. 73.7 and 60.2% women in South Africa and Zimbabwe, respectively started their pregnancy with BMI above normal (BMI ≥ 25) whereas the prevalence of underweight was virtually non-existent. The majority of women had inadequate intakes of micronutrients. Considering food and beverage intake only, none of the micronutrients measured achieved the estimated average requirement. Around 60% of pregnant women reported taking folic acid or iron supplements in South Africa, but almost none did so in Zimbabwe.

**Conclusion:**

We found a high prevalence of overweight and obesity and high micronutrient intake inadequacy in pregnant women who had the previous pregnancy complicated with pre-eclampsia. The obesity figures and micronutrient inadequacy are issues of concern that need to be addressed. Pregnant women have regular contacts with the health system; these opportunities could be used to improve diet and nutrition.

**Trial registration:**

PACTR201105000267371. Registered 06 December 2010.

## Background

Nutrition status of women before and during pregnancy is one of the main contributors to pregnancy outcomes and early child health [1]. In many low and middle-income countries undernutrition and overnutrition coexist in the same population [2]. Obesity is increasing while micronutrient deficiencies still persist, particularly in the most vulnerable groups such as women and children [3]. Consequently, women start pregnancy with higher risks to develop complications such as pre-eclampsia, gestational diabetes mellitus, gestational hypertension, depression, fetal macrosomia, stillbirth, preterm birth, birth by caesarean section and infant mortality [4–9]. In addition, high maternal body mass index (BMI) has also been associated with delayed breastfeeding, weight retention and in women with gestational diabetes, a higher risk of developing chronic diseases [5]. Inter-pregnancy interval is also an important factor that may influence maternal availability of nutrients, especially in those populations with existing micronutrient deficiencies [10].

Interest in pre-conceptional interventions to reduce risk factors during pregnancy is growing, although their effectiveness on pregnancy outcomes is less certain [11]. Current WHO Guidelines on antenatal care recommend supplementation with iron and folic acid to all pregnant women, and with calcium and vitamin A to women in specific areas with a high prevalence of deficiency [12]. In populations where calcium intake is low, the WHO recommends supplementation with 1.5–2.0 g elemental calcium/day from 20 weeks´ gestation until the end of pregnancy for the prevention of pre-eclampsia. The WHO Guidelines also report that women receiving counselling on diet and/or exercise are less likely to experience excess weight gain during pregnancy, although the evidence on the impact of other pregnancy outcomes is less certain [12].

In South Africa, the National Health and Nutrition Examination Survey (SANHANES-1) reported a prevalence of overweight (BMI ≥ 25 kg/m2) and obesity (BMI ≥ 30) in women of 24.8 and 39.2%, respectively in 2012 [13]. Data from Zimbabwe in 2000 shows a prevalence of overweight and obesity in women of 17.4 and 5.7% respectively [14]. However information on overweight and obesity rates as well as dietary intake during pregnancy is scarce in these countries. Nutrient and supplement intake information would be important to better plan and tailor interventions to improve pregnancy outcomes [10].

We conducted a randomized controlled trial to evaluate the effect of pre-pregnancy calcium supplementation on the incidence of recurrent pre-eclampsia (Calcium and Pre-eclampsia: CAP trial) [15]. This was a multi-country trial conducted in South Africa, Zimbabwe and Argentina. This manuscript presents the results of a sub-analysis of the CAP trial with the aim of describing the nutritional status of women from South Africa and Zimbabwe that became pregnant during the CAP trial. More specifically, we aimed to describe levels of overweight and obesity before and during pregnancy, and the adequacy of macronutrient and micronutrient intake during pregnancy.

## Participants and methods

This is a nested prospective observational study of women from South Africa and Zimbabwe recruited in the CAP trial [15]. The CAP trial is a multi-centre randomized, double-blind placebo-controlled clinical trial with the objective to determine whether calcium supplementation before conception and during the first half of pregnancy reduces the incidence of recurrent pre-eclampsia more effectively than supplementation starting at 20 weeks, which is the current WHO recommendation. In the CAP trial, non-pregnant women with history of pre-eclampsia or eclampsia in their most recent pregnancy were invited to participate as they are at higher risk of developing pre-eclampsia in subsequent pregnancies. Once admitted in the trial, participants were required to attend study sites every 12 weeks for follow up until pregnancy occurred. Pregnant women were followed up throughout their pregnancy and trial visits were scheduled at 8, 20 and 32 weeks´ gestation. Eligible women were randomized to receive either 500 mg of elemental calcium daily or placebo from recruitment and blinded supplementation continued while participants were non-pregnant or until 20 weeks’ gestation. From 20 weeks’ gestation, all participants received calcium supplements in compliance with WHO guidelines [16]. The CAP trial started in 2011 and recruitment was completed in September 2016.

### Settings and study population

Participants were recruited from government secondary or tertiary urban referral hospitals with large obstetric units serving urban and rural populations. The maternity and obstetric units included in the CAP trial were located in Cape Town (1), East London (2) and Johannesburg (1) in South Africa; and in Harare (2), Zimbabwe. Women were eligible for the CAP trial if they had pre-eclampsia or eclampsia in their most recent pregnancy, if they were not pregnant but in a sexual relationship, not using contraception and if they gave informed consent. For admission we reviewed the participant clinical records and accepted the clinical evaluation of pre-eclampsia or eclampsia reported there. Exclusion criteria included: less than 18 years of age; chronic hypertension with persistent proteinuria; calcium supplement intake; and history or symptoms of urolithiasis, renal disease or parathyroid disease [15]. For a complete list of eligible criteria please refer to the published protocol [15]. In this analysis, we included women recruited in the CAP trial who became pregnant and reached 20 weeks´ gestation between March 2013 to March 2016.

### Anthropometric assessment and clinical data collection

Variables used for this sub-study included: age, height, pre-pregnancy weight, number of previous pregnancies and date of birth of last pregnancy complicated with pre-eclampsia. This data were collected at admission. For women who became pregnant, weight during pregnancy was recorded at 8, 20 and 32 weeks´ gestation during the scheduled trial follow-up visits. Research nurses specially trained for the CAP trial assessed all anthropometric, clinical and dietary variables at admission and during follow up visits at each participating site.

Body weight was measured to the nearest 0.1 kg in light clothing and without shoes. Height was measured to the nearest 0.1 cm and without shoes using a stadiometer. Scales and stadiometers were those provided by each hospital and remained the same throughout the study. The Manual of Operations and the Standard Operating Procedures (SOPs) provided clear instructions on how women should be weighted and measured.

Pre-pregnancy BMI was calculated as weight (kg) divided by the square of the body height (m) using measurements recorded at admission. Women were classified according to the WHO BMI standards for adults, defined as underweight (BMI < 18.5), normal (18.5 ≤ BMI < 25), overweight (BMI ≥ 25), or obese (BMI ≥ 30) [17].

Gestational weight gain was calculated by subtracting the weight at 8 weeks´ gestation from the weight at 32 weeks´ gestation, since the participant’s weight at delivery was not assessed.

### Dietary assessment

The dietary intake of participants was assessed at 20-weeks’ gestation using a triple pass 24-h dietary recall adapted from the method developed by Nelson M. team from the King’s College London after it was piloted in South Africa and Zimbabwe [18]. The 24-h recall is a guided interview to assess food intake of the previous day. CAP trial research nurses were trained in-site in March 2013 to administer the triple pass 24-h recalls and to use the Dietary Assessment Education Kit (DAEK) to assist with the portion size estimation [19]. Xhosa and Zulu translators were trained at the sites that required them.

Reported food intakes from the 24-h recall were entered and analysed using the Food Finder III computer program, provided by the South African medical research council (SAMRC) to obtain daily energy and nutrient intakes for each participant. If properly conducted, a single day 24-h dietary recall is a reliable method to assess individual intake on one day and can be used to estimate a population mean [20]. However, as food and nutrient intakes have a wide day-to-day variability, data obtained from one single day is not sufficient to describe the usual intake or to assess the proportion of individuals with intakes below certain thresholds (e.g. below requirements). Statistical models have been developed to better estimate usual nutrient intakes in a population by adjusting for within-individual intake variability [21]. Therefore, in order to estimate the proportion of women with intakes below requirements we used the Intake Distribution Estimation software (PC-SIDE, version 1.0, 2003; Department of Statistics, Iowa State University, Ames) that requires a sample of at least 50 dietary assessments that are repeated on a non-consecutive day to the first assessment [22]. For these purposes, a second 24-h dietary recall assessment was administered in a subsample of women on a non-consecutive day after the first 24-h recall. Energy and nutrient intake distributions from the single 24-h recall were thus adjusted by within-person variance obtained from the second 24-h recall assessment and by interview weekday to estimate usual nutrient intake and to calculate the proportion of women with intakes below requirements.

The Estimated Average Requirement (EAR) of carbohydrates and each micronutrient as recommended by the Institute of Medicine (IOM) for pregnant women was used as the cut-off point to assess adequacy of nutrient intake [23]. The Estimated Energy Requirement (EER) for pregnant women during the second trimester was calculated for each participant using the age, weight and height at admission and according to the Dietary Reference Intake (DRI) formula for adult women [23]. As data of physical activity was not collected the value for sedentary lifestyle was used conservatively. Energy intake obtained from the first 24-h recall assessment was divided by the EER then normalized using PC-SIDE [23, 24]. The 80% of EER during pregnancy suggested by Goldberg was used to calculate the plausibility of energy intake [25]. Protein adequacy was calculated using the EAR of 0.88 g per kg of body weight, using weight at 20 weeks´ gestation [23].

A specific questionnaire was also included to investigate supplement intake during pregnancy. Women were asked about frequency and dose of the supplements and medicines. Trial supplementation was not computed in the dietary assessment of this sub-study as it was the intervention being tested and not, otherwise, part of the diet of this group of women.

### Statistical analysis

Categorical values were described using percentages and numerical variables using means and standard deviations (SD). Statistical data analyses were performed using the SPSS 23.0 software package (IBM, New York, NY, USA). Dietary intake variables were log transformed and tested for normality using the Anderson-Darling statistical test using the PC-SIDE software. The software performs three steps: adjustments for weekday; transformation to normality using power transformation; and estimation of within-person variance using an error measurement model.

### Ethics

Ethical approval was obtained from appropriate national and institutional ethics review bodies as applicable for each study site, and all participants provided informed written consent. The study was approved by the Research Project Review Panel of the UNDP/UNFPA/ UNICEF/WHO/World Bank Special Programme of Research, Development and Research Training in Human Reproduction at the Department of Reproductive Health and Research of WHO, and the WHO Research Ethics Review Committee, Geneva, Switzerland.

Data management procedures were compliant with good clinical practice (GCP) [26].

## Results

**Fig. 1:**
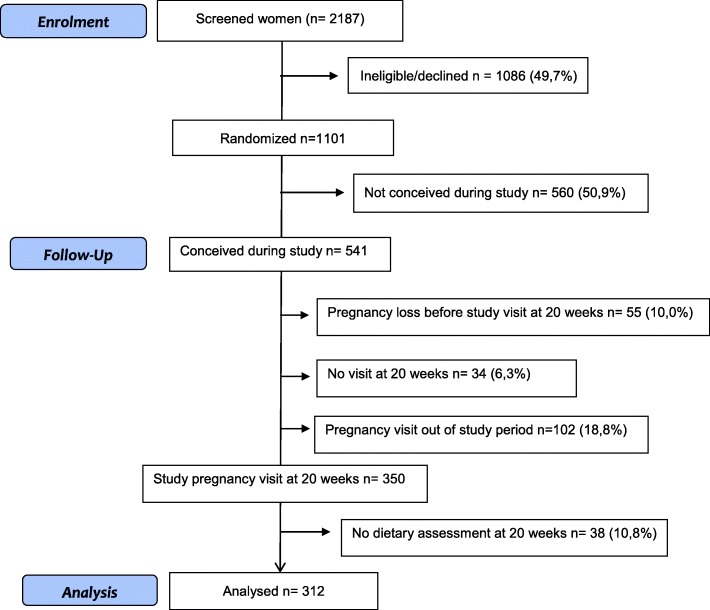
Flow chart

A total of 2187 women were screened in South Africa and Zimbabwe during the sub-study period (March 2013 to March 2016) and 1101 (50.3%) were eligible and accepted to participate (Fig. 1). Of the 1101 participants randomized, 541 (49.1%) became pregnant of whom, 55 (10.0%) had a miscarriage or a pregnancy termination before 20 weeks´ gestation, 34 did not attend to the pregnancy visit at 20 weeks’ gestation, and 102 (18.8%) completed the visits at 20 weeks´ gestation outside the sub-study period. A total of 350 women were eligible for this sub-study, however 38 (10.8%) missed the dietary assessment interview. Thus, we present the results of 312 women that completed the dietary assessment at the 20 weeks´ gestation visit. Of these women 224 (71.8%) were from South Africa and 88 (28.2%) from Zimbabwe. Repeated dietary assessments were obtained from 107 (34.3%) women, 79 from South Africa and 28 from Zimbabwe.

At the time of the assessment, women from South Africa had been in the study an average of 12.5 (SD ±7.4) months and women from Zimbabwe 13.1 (SD ±7.4) months. Their inter-pregnancy interval was 24.5 (SD ± 22.5) months in South Africa and 30.3 (SD ±23.7) months in Zimbabwe (Table 1).

**Table 1:** Participant Characteristics

	All		South Africa		Zimbabwe	
	*n* = 312	%	*n* = 224	%	*n* = 88	%
Age (years)						
Less than 20	1	0.3	0	0	1	1.1
20 to less than 35	255	81.7	182	81.3	73	83
35 and older	56	17.9	42	18.8	14	17.9
Parity						
1	121	38.8	93	41.5	28	31.8
2	111	35.6	79	35.3	32	36.4
3 or more	79	25.3	51	22.8	28	31.8
Missing	1	0.3	1	0.4	0	0
Months in study from admission to 20 weeks´ gestation						
Mean (sd)	312	12.5 (7.4)	224	13.1 (7.4)	88	11.0 (7.4)
Less than 6 month	94	30.1	60	26.8	34	38.6
6 to less than 12 month	88	28.2	59	26.3	29	33.0
12 to less than 24 month	97	31.1	79	35.3	18	20.5
24 or more month	32	10.3	25	11.2	7	8.0
Missing	1	0.3	1	0.4	0	0
Months since last birth with PE to 20 weeks´ gestation						
Mean (sd)	312	24.5 (22.5)	224	30.3 (23.7)	88	27.4 (19.0)
Less than 6 month	9	2.9	7	3.1	2	2.3
6 to less than 12 month	54	17.3	39	17.4	15	17.0
12 to less than 24 month	94	30.1	62	27.7	32	36.4
24 or more month	135	43.3	102	45.5	33	37.5
Missing	20	6.4	14	6.3	6	6.8
Anthropometric variables						
Height at admission - mean (sd)	286	160.3 (6.4)	202	159.9 (6.4)	84	161.1 (6.4)
Weight at admission - mean (sd)	302	76.1 (17.4)	217	78.9 (18.2)	85	69.0 (12.6)
Weight at week 8 of gestation - mean (sd)	251	77.1 (16.9)	173	80.2 (17.7)	78	70.2 (12.4)
Weight at week 20 of gestation - mean (sd)	300	80.2 (17.5)	215	83.5 (18.1)	85	71.9 (12.6)
Weight at week 32 of gestation - mean (sd)	224	84.0 (17.0)	158	88.2 (17.5)	66	74.0 (10.1)
Mean BMI at admission - mean (sd)	283	29.6 (6.3)	201	30.7 (6.6)	82	26.8 (4.5)
Body Mass Index at admission						
Underweight (BMI < 18.5 kg/m2)	3	1.0	2	0.9	1	1.1
Normal (18.5 BMI < 25 kg/m2)	62	19.9	34	15.2	28	31.8
Overweight (25 BMI < 30 kg/m2)	95	30.4	62	27.7	33	37.5
Obesity I (30 BMI 35 kg/m2)	75	24.0	58	25.9	17	19.3
Obesity II (35 BMI 40 kg/m2)	32	10.3	30	13.4	2	2.3
Obesity III (BMI > 40 kg/m2)	16	5.1	15	6.7	1	1.1
Missing	29	9.3	23	10.3	6	6.8

### Clinical characteristics

At recruitment, the mean age of women in this sub-study was 29.2 years (SD ± 5.2) in South Africa and 29.3 (SD ± 4.8) in Zimbabwe. Parity was three or more in about 25% of the women (22.8 and 31.8% in South Africa and Zimbabwe, respectively). The mean height was 159.9 cm (SD ±6.4) in South Africa and 161.1 cm (SD ±6.4) in Zimbabwe; and the mean weight before pregnancy was 78.9 kg (SD ±18.2) in South Africa and 69.0 kg (SD ±12.6) in Zimbabwe. The prevalence of overweight was 27.7% in South Africa and 37.5% in Zimbabwe while the prevalence of any degree of obesity was 46.0% in South Africa and 22.7% in Zimbabwe (Table 1). In total, 73.7 and 60.2% women in South Africa and Zimbabwe, respectively entered the trial with BMI above normal. On the other hand, the prevalence of underweight was virtually non-existent in both countries.

### Gestational weight gain

We found that women who were initially classified according to their BMI as normal weight had gained from 8 to 32 weeks’ gestation an average 8.9 kg (SD ± 4.4) in South Africa and 7.4 kg (SD ± 3.3) in Zimbabwe. Those classified as overweight had gained 7.8 kg (SD ± 4.5) in South Africa and 5.8 kg (SD ±3.8) in Zimbabwe and those classified as obese had gained 5.9 kg (SD ± 6.2) in South Africa and 3.1 kg (SD ± 4.1) in Zimbabwe.

### Diet

#### Macronutrients

The average total daily energy intake was 1765.6 kcal (SD ±346.6) in South Africa and 1827.9 (SD ±303.9) in Zimbabwe. Average daily carbohydrate, fat and protein intakes were 230.8 (SD ± 57.5) grams, 59.1 (SD ± 6.4) grams and 54.7 (SD ±7.8) grams in South Africa and 213.6 (SD ± 22.3) grams, 76.4 (SD ± 24.8) grams and 52.9 (SD ± 25.8) grams in Zimbabwe, respectively (Table 2). Most (54.4%) of total energy intake came from carbohydrates, 27.8% from fats and 12.6% from proteins in South Africa while the percentages in Zimbabwe were 48.0, 36.38 and 11.26% respectively.

**Table 2:** Usual intake from foods and beverages, excluding supplements, estimated using repeated 24-h recalls in 312 women and repeated in a sub-sample of 107 women, and percentage of women with usual intake below Estimated Average Requirement (EAR)

Estimated Daily Usual Intake^b^	South Africa	Zimbabwe	EAR^a^	% women with intakes below EAR	
	*n* = 224	*n* = 88		South Africa	Zimbabwe
Energy (kcal)	1765.6 (346.6)	1827.9 (303.9)	NA^c^	NA	NA
EER Estimated Energy Requirement (%)	0.88 (0.20)	0.93 (0.14)	0.8	37.7	19.1
Protein (g)	54.7 (7.8)	52.9 (25.8) ^d^	NA	NA	NA
Protein (g/kg)	0.82 (0.43)	0.79 (0.04)	0.88	71.1	98.3
Carbohydrates (g)	230.8 (57.5)	213.6 (22.3)	135	02.9	0.00
Total Sugars (g)	45.4 (19.4)	35.4 (10.7)	NA	NA	NA
Fats (g)	59.1 (6.4)	76.4 (24.8)	NA	NA	NA
Calcium (mg)	441.0 (97.7)	360.5 (171.4)	800	99.9	97.6
Iron (mg)	9.9 (5.3)	7.9 (1.7)	22	96.9	100.0
Folate (mcg)	253.2 (69.1)	240.6 (46.2)	520	99.9	100.0
Mg (mg)	239.6 (51.1)	262.5 (38.8)	290	83.9	77.3
Zn (mg)	7.3 (1.7)	6.4 (1.6)	9.5	90.4	95.7
Se (mcg)	37.9 (4.3)	37.5 (11.8)	49	99.1	83.7
Riboflavin (mg)	1.3 (0.6)	0.8 (0.5)	1.2	54.0	84.0
Niacin (mg)	13.0 (3.8)	13.0 (3.7)	14	63.8	64.9
Vitamin C (mg)	82.4 (45.1)	109.80 (41.8)	70	46.6	15.6
Vitamin E (mg)	10.1 (3.8)	26.4 (9.1)	12	73.5	03.7

^a^*EAR* Estimated average requirement

^b^Usual intake was obtained using the IOWA methodology

^c^*NA* not applicable)

^d^This value represents the mean of the first interview

Average daily intake of total sugars was 45.6 (SD ± 40.9) grams in South Africa and 34.5 (SD ±26.0) grams in Zimbabwe representing 10.6 and 7.5% of the total energy intake. The majority of women in both countries had lower than recommended protein intake and a higher intake of carbohydrates (Table 2).

#### Micronutrients

Adjusted usual nutrient intakes (from food and beverage, excluding supplements) are presented in Table 2. Micronutrient inadequacy was highly prevalent in both countries. Almost all women in South Africa had inadequate intakes of folate, calcium, iron and selenium, while the majority also had inadequate intakes of magnesium, zinc, niacin and vitamin E. More than half of the women had inadequate intakes of riboflavin while less than half had inadequate intake of vitamin C. All women in Zimbabwe had inadequate dietary intakes of iron and folate; the majority also had inadequate intakes of calcium, magnesium, zinc, selenium and riboflavin. The intake of vitamin C and E was however, adequate in the majority of women from Zimbabwe. (Table 2).

#### Supplements

In South Africa, 62.9% of women (141) reported taking 5 mg of folic acid supplements, 57.1% (128) reported taking iron supplements with doses ranging between 75 to 400 mg; 24.1% (54) reported taking vitamin C with doses ranging between 100 to 250 mg daily and 9.8% (22) reported taking vitamin B complex. At 20 weeks of pregnancy, women reported taking these supplements for a mean period of 2.1 to 2.7 months. Other supplements reported include calcium gluconate, magnesium sulphate and copper sulphate. Most of the supplements were provided by the hospital. On the other hand, a total of 29 (12.9%) women reported taking multivitamins for a mean period of 1 to 2 months, which are not provided by the hospitals.

In Zimbabwe, only one woman reported taking iron supplements during pregnancy. No other types of supplements intake were reported what so ever.

## Discussion

This study shows that a high proportion of women whose previous pregnancy was complicated by pre-eclampsia in hospitals from South Africa and Zimbabwe started their subsequent pregnancy overweight or obese (73.7% in South Africa and 60.2% in Zimbabwe). In fact, obesity affected about 1 in 4 women in Zimbabwe, and as many as 1 in 2 women in South Africa were obese. Furthermore, at 20 weeks´ gestation more than 90% of these women had intakes of micronutrients, like iron, calcium, folate and zinc below requirements.

Overweight and obesity problems have already been reported in these countries. The prevalence of overweight or obese women found in our study is higher than the 64% that has been reported for South Africa by the SANHANES-1 and the 54.9 and 25% that the WHO Global Database on Body Mass Index reports for South Africa in 2004 and Zimbabwe in 2006 respectively, but in line with other studies conducted in South Africa that reported 69% [27–29]. The fact that we only included women who had a previous pregnancy complicated with pre-eclampsia could contribute to the higher overweight or obesity prevalence in our study population [14, 15]. A link between obesity and hypertensive disorders of pregnancy has been reported in the literature. A systematic review concluded that for every 5 to 7 kg/m^2^ increase in BMI, the risk of developing pre-eclampsia doubles which confirms the relevance and critical importance of developing and implementing special efforts to control the BMI of these women before they become pregnant [30].

We found that women with normal BMIs’ at 8 weeks´ gestation, compared to those with higher BMI, gained more weight at 32 weeks, which is in accordance to recommendations [31]. However, as we only assessed weight up to 32 weeks´ gestation and most gestational weight gain occurs after 20 weeks´ gestation, we would expect that many of these women would exceed the Institute of Medicine recommendations. In our study, women classified as normal weight gained from 8 to 32 weeks’ gestation an average 8.9 kg (SD ± 4.4) in South Africa and 7.4 kg (SD ± 3.3) in Zimbabwe of the 11.5 to 16 kg recommended for this group. Those classified as overweight gained 7.8 kg (SD ± 4.5) in South Africa and 5.8 kg (SD ±3.8) in Zimbabwe of the 7 to 11.5 kg recommended for this group and those classified as obese gained 5.9 kg (SD ± 6.2) in South Africa and 3.1 kg (SD ± 4.1) in Zimbabwe of the 5 to 9 kg recommended for this group [12]. Programmes and interventions to reduce obesity before pregnancy and control weight gain during pregnancy would be advisable in view of the findings of this analysis.

The energy intake we report is similar to those reported for women in the US National Health and Nutrition Survey in 2010–2011 where the prevalence of overweight and obesity are also higher than 70% [32, 33]. According to Goldberg, if the estimated usual intake is below 80% of the estimated average requirement for a person, this would imply under-reporting. In our study this would imply 37.7% of underreporting in South Africa and 19.1% in Zimbabwe [25]. Fat intake as a percentage of total energy was within the recommended range of 20 to 35% of total energy in South Africa (27.8%), but slightly higher than recommended in Zimbabwe (36.4%). The high fat intake in Zimbabwe was due to a higher intake of polyunsaturated fats. In both countries, carbohydrates and protein intake as a percentage of total energy were within the recommended ranges of 46–65% and 10–35% respectively [23]. However, the intake of sugar in South Africa was slightly above the recommended maximum of 10% of total energy. Considering recommended grams of macronutrients, the women from both countries had mostly adequate total grams of carbohydrate intakes, but the majority had inadequate grams per kilograms of protein intake. It is thus important that interventions should focus on increasing the intake of affordable protein sources and decreasing sugar intake.

The prevalence of inadequate micronutrient intake from food sources was high in both countries. For the most basic micronutrients like iron, calcium, folate and zinc, the percentage of women below requirements was above 90% in both countries. The most common supplements taken in South Africa were folic acid, ferrous sulphate, and vitamin C, all issued by the hospital. There is a policy in South Africa to supplement pregnant women with 5 mg of folic acid and 200 mg of ferrous sulphate (equivalent to 40 mg elemental iron) daily, which allowed those women taking the supplements to reach the recommendations [34, 35]. The elemental iron supplementation provided by this policy is in accordance with the WHO guidelines for antenatal care, however folic acid supplementation is more than 10 times the recommended amount [12]. On the other hand, vitamin C is not recommended, as there is no evidence of an impact on birth outcomes. In contrast, only one woman in Zimbabwe reported taking supplements and this may be due to the fact that there is no policy to provide supplements during pregnancy in Zimbabwe and very few women bought commercial supplements. The inadequate intakes are worrying as iron and folic acid supplementation are known to prevent anaemia, puerperal sepsis, low birth weight and preterm birth; folate to prevent neural tube defects, calcium supplementation in areas with low calcium intake in order to reduce risk of pre-eclampsia; vitamin A in deficient areas to prevent night blindness. Regarding the diet a higher intake of vitamin E is usually related to higher intakes of oils and fats whereas vitamin C indicates an increased intake of fruits and vegetables [36, 37].

### Strengths

Strengths of this study include the standardised procedures used throughout the trial in all sites. Women in this study had close follow up from the same research team before and during pregnancy.

The 24-h recall dietary assessment method used is subject to less recall bias than other dietary assessment methods, such as diet histories or food frequency checklists [38]. Major advantages of using 24-h recalls are that high literacy of the respondent is not required and that inter-observer differences are minimised [18]. On the other hand, food frequency questionnaires usually require use of generic memory and higher numeracy skills in the population interviewed to quantify average food intakes over a period of time [39].

### Limitations

We did not use the same scales to measure weight across sites, as weight was not the main outcome of this trial. However, women were assessed with the same scale throughout the study at each participating site.

We did not use any technique to corroborate energy intake, however under-reporting has been described in the literature in women especially in those with high BMI [40].

Food data from both countries was analysed using the SAMRC-Food composition database as there is not a local food composition database in Zimbabwe. There were a few cases where foods reported during the assessment were not found in the database, and they were added as the most similar item in terms of macronutrients and calcium content. Nevertheless, this was only in a few cases and we believe it cannot affect the main results of this sub-study. Although not having a database for Zimbabwe might be a limitation, it prevents showing differences that are related to errors of the food composition tables rather than of the nutrient intake [41].

The IOWA methodology to estimate usual intake requires at least 50 repeated interviews, we obtained repeated 24-h recall for 107 women, however we obtained fewer than 50 repeated interview for Zimbabwe so the estimation might not be as accurate as for South Africa.

## Conclusion

We found a high prevalence of overweight and obesity and high prevalence of inadequate intakes of protein and micronutrients in pregnant women who had a previous pregnancy complicated with pre-eclampsia. Although this group is not representative of the general population of pregnant woman, taking into account the increasing prevalence of overweight and obesity worldwide among young age groups, the obesity and micronutrient inadequacy figures reported in this study are issues of concern that need to be addressed so that maternal and perinatal outcomes are improved [42, 43]. Noticing the differences found in both countries regarding BMI and nutrient intake it would be interesting to explore the reasons behind as it could help to tackle the problem.

Supplement intakes during pregnancy seem to be essential for these groups of women to achieve requirements of key micronutrients. Policies should be reinforced and reviewed according to the most recent evidence. Pregnancy is a period when women may have more regular contacts with the health system and, if health care services were integrated for mothers and babies, these regular contacts could be maintained after delivery to improve maternal health [44]. These opportunities could be used to deliver dietary and nutritional interventions to high-risk women to improve the outcomes of future pregnancies [45]. Furthermore, taking into account that women with a history of pre-eclampsia are at higher risk of developing cardiovascular disease later in life, pregnancy and postnatal periods could be an ideal time for preventing future health complication from a young age.

## Abbreviations

BMI: Body mass index
CAP: The calcium and pre-eclampsia study
DRI: Dietary reference intake
EAR: Estimated average requirement
EER: Estimated energy requirement
IOM: Institute of Medicine
SAMRC: South African Medical Research Council
SANHANES-1: National Health and Nutrition Examination Survey
WHO: World Health Organization
